# Risk of caesarean section after induced labour: do hospitals make a difference?

**DOI:** 10.1186/1756-0500-6-214

**Published:** 2013-05-28

**Authors:** Cristina Teixeira, Sofia Correia, Henrique Barros

**Affiliations:** 1 Institute of Public Health, University of Porto, Porto, Portugal; 2 Polytechnic Institute of Bragança, Bragança, Portugal; 3 Department of Clinical Epidemiology, Predictive Medicine and Public Health, University of Porto Medical School, Porto, Portugal

**Keywords:** Caesarean section, Health care services, Induction of labour, Women’s health

## Abstract

**Background:**

There is a well-known relationship between induced labour and caesarean rates. However, it remains unknown whether this relationship reflects the impact of more complex obstetric conditions or the variability in obstetric practices. We sought to quantify the independent role of the hospital as a variable that can influence the occurrence of caesarean section after induced labour.

**Methods:**

As part of the Portuguese Generation XXI birth cohort, we evaluated 2041 consecutive women who underwent singleton pregnancies with labour induction, at five public level III obstetric units (April 2005-August 2006). The indications for induction were classified according to the guidelines of the American and the Royal Colleges of Obstetricians and Gynaecologists. Poisson regression models were adjusted to estimate the association between the hospital and surgical delivery after induction. Crude and adjusted prevalence ratios (PR) and a 95% confidence interval (95% CI) were computed.

**Results:**

The proportion of women who were induced without formal clinical indications varied among hospitals from 20.3% to 45.5% (p < 0.001). After adjusting for confounders, the risk of undergoing a caesarean section after induced labour remained significantly different between the hospitals, for the cases in which there was no evident indication for induction [the highest PR reaching 1.86 (95% CI, 1.23–2.82)] and also when at least one such indication was present [1.53 (95% CI, 1.12–2.10)]. This pattern was also observed among the primiparous cephalic term induced women [the highest PR reaching 2.06 (95% CI, 1.23–2.82) when there was no evident indication for induction and 1.61 (95% CI, 1.11–2.34) when at least one such indication was present].

**Conclusions:**

Caesarean section after induced labour varied significantly across hospitals where similar outcomes were expected. The effect was more evident when the induction was not based on the unequivocal presence of commonly accepted indications.

## Background

The past few decades have witnessed a worldwide increase in caesarean rates [1]. This increase has resulted from evidence-based recommendations on how to handle certain conditions, such as anomalous foetal position [2], major placental abruption [3], placenta praevia [4] and prolapsed cord [5]; however it is mainly the consequence of a growing number of women presenting at labour with uterine scars [6], delivering at advanced ages [7], or demanding surgical delivery [8]. Still, the increased frequency of obstetric interventions, such as induction of labour, appears to have contributed to the current trends in caesarean rates [9].

The increased risk of caesarean section after induced labour is well documented [10-19], but such obstetric intervention is considered justified when the benefits of prompt delivery outweigh the consequences of a caesarean section. Conditions such as post-term pregnancy, hypertensive disorders, intrauterine growth restriction and diabetes are commonly accepted indications for induction [20-22]. However, there is evidence for an increase in the frequency of labour induction without any such agreed upon indication [23,24]. This situation might lead to unnecessary caesarean deliveries and, consequently to a high risk of adverse outcomes for the mother [25-28] and the child [29-31]. In the absence of a well-established clinical indication, the contribution of labour induction to caesarean rates among low-risk nulliparous women can approach 20% [17]. Additionally, labour induction in itself increases in-hospital pre-delivery and labour time and costs [10,12] beyond those that are related to surgery [32].

Labour induction rates present a wide country variation [33], but variability is also present among obstetric units in the same geographic region [15,34,35] or practitioners within the same hospital [13,34]. This variability is the result of differences in the case mix, obstetric protocols or the judgment of the individual physician regarding the appropriateness of obstetric interventions [13]. Differences that are not justified by the case mix reveal modifiable attitudes in clinical practice that might result in health gains. Comparing hospitals with similar funding resources, obstetric care levels and neonatal support is a particularly interesting approach to address this issue.

With the present investigation, we sought to identify how these factors affected the occurrence of caesarean section after induced labour by studying five Portuguese public hospitals that offered the highest levels of obstetric and neonatal care (level III) free of charge to childbearing women and their offspring.

## Methods

The Portuguese health care system provides prenatal, obstetric, neonatal and pediatric services free of charge for all childbearing women (citizen or foreign-born) and their children. There is also a market supply of private health care services. Although almost 40% of Portuguese pregnant women choose to have prenatal care under private physicians, 90% of them deliver in public hospitals. Still the majority of these deliveries take place in obstetric units level III that offer the highest level of obstetric and neonatal care.

The participants of the present study were recruited in five public hospitals level III, while assembling a birth cohort in Porto Metropolitan Area, in the north of Portugal (Generation XXI). Between April 2005 and August 2006, 70% of all pregnant women delivered at those five public hospitals were invited as participants on the basis of “first come first served” and only 8% of those invited refused to participate. This approach allowed a representative sample. The final sample comprised 8495 women who delivered live infants (>24 weeks); there were 8351 singleton pregnancies. Information on patient social and demographic characteristics, obstetric and gynaecological history, lifestyles and current pregnancy events was obtained using a structured questionnaire. Individual interviews were performed 24 to 72 hours after delivery by trained interviewers. Information on pregnancy complications, delivery circumstances and data on newborn characteristics were abstracted from patient medical records.

This study included only the 2041 women (24.4%) who delivered a singleton live infant after induced labour (Table 1). The mode of delivery was divided by vaginal or caesarean section deliveries. The covariates considered were maternal age (continuous variable), education level (> = 6, 7–9, 10–12 and >12 years of schooling), woman´s country of origin (Portuguese, European other than Portuguese, African, South American and Other), parity and previous caesarean section (primiparous, multiparous with no previous caesarean section and multiparous with previous caesarean section) body mass index (BMI, <25.0, 25.0–29.9 and > =30 Kg/m^2^), type of antenatal care (only public services or at least one visit at private services), foetal presentation (cephalic or non-cephalic presentation), gestational age (<37, 37, 38, 39, 40 and > =41 weeks), epidural anaesthesia (yes or no) and the diagnosis of at least one indication for labour induction. Following the American College of Obstetricians and Gynaecologists (ACOG), Royal College of Obstetricians and Gynaecologists (RCOG) and local guidelines [20-22], we assumed the following main indications for labour induction: the presence of premature rupture of membranes (PROM), post-term pregnancy (> = 41 weeks), diabetes (gestational or pre-gestational), hypertension (chronic or gestational), maternal diseases that could demand prompt delivery (chronic pulmonary, renal, or hepatic diseases, gestational pyelonephritis, gestational cholestasis, and antiphospholipid syndrome), foetal growth restriction, macrosomia, amniotic fluid disorders, and isoimmunisation. Foetal growth restriction and macrosomia were proxied as small or large by gestational age, respectively, using the curves from a population-based study [36]. Based on the information retrieved from medical charts, we considered three groups of women according to the number of these indications (none, one and two or more indications). Because few women were in the latter group (n = 385), further analysis considered only two groups (none or at least one indication).

**Table 1:** Labour onset by hospital

**Hospital**	**1**	**2**	**3**	**4**	**5**
	**n = 1984**	**n = 1404**	**n = 884**	**n = 2040**	**n = 2039**
**Labour Onset**					
Spontaneous	1223 (61.6)	1063 (75.7)	362 (41.0)	1303 (63.9)	1257 (61.6)
Induced	455 (22.9)	237 (16.9)	369 (41.7)	468 (22.9)	512 (25.1)
Caesarean before labour	235 (11.8)	93 (6.6)	119 (13.5)	235 (11.5)	221 (10.8)
Not classifiable	71 (3.6)	11 (0.8)	34 (3.8)	34 (1.7)	49 (2.4)

Poisson regression models were fitted to estimate the individual level association between hospital and the caesarean section after induction [37]. Adjusted prevalence ratio (PR) values with 95% confidence intervals (95% CI) were computed. A baseline model was fitted containing the hospital as independent variable and using hospital with the lower caesarean prevalence as reference. All covariates were individually checked using manual forward addition and backward deletion and kept on final model if they changed the PRs of caesarean section after labour induction by hospital at least 10%. Interaction between the independent variables was also tested and stratified analyses were performed accordingly. In order to strength the analysis we also performed such analyses in a standard group: the primiparous term cephalic pregnant women. We presented also the proportion of women with diagnosis of failed induction and/or poor progression in labour.

Statistical analysis was performed using IBM SPSS Statistics (version 19.0) and the level of significance was set at p < 0.05.

The study was approved by the Ethics Committee of the University of Porto Medical School/Hospital S. João and written informed consent was obtained from each participant.

## Results

As shown in Table 1, in the five hospitals, the proportion of women that underwent induction of labour ranged between 16.9% and 41.7% (p < 0.001).

Table 2 presents the demographical and clinical characteristics of the women who underwent labour induction (n = 2041). The hospitals presented significantly different distributions of women regarding maternal age (p < 0.001), educational level (p < 0.001), BMI (p = 0.010), type of antenatal care (p < 0.01), foetal presentation (p = 0.002) and gestational age (p < 0.001). The proportion of women with no clinical indications for induced labour varied from 20.3% to 45.5% (p < 0.001). The overall prevalence of caesarean section was 41.6%, and this prevalence varied significantly across the hospitals (between 32.5% and 48.4%, p < 0.001).

**Table 2:** Demographic, clinical and health care characteristics of induced women by hospital

**Hospital**	**1**	**2**	**3**	**4**	**5**	**p-value**
	**n = 455**	**n = 237**	**n = 369**	**n = 468**	**n = 512**	
	**n (%) or mean ± standard deviation**					
**Maternal age (years)**	29.4 ± 5.86	30.5 ± 5.44	30.3 ± 4.98	29.6 ± 4.81	28.9 ± 5.59	<0.001
**Education level (years)**						
= < 6	162 (35.6)	68 (28.7)	77 (20.9)	101 (21.6)	147 (28.7)	<0.001
7 – 9	114 (25.1)	51 (21.5)	86 (23.3)	97 (20.7)	181 (35.4)	
10 – 12	83 (18.2)	49 (20.7)	100 (27.1)	125 (26.7)	103 (20.1)	
> 12	92 (20.2)	68 (28.7)	106 (28.7)	143 (30.6)	78 (15.2)	
no information (%)	0.9	0.4	0.0	0.4	0.5	
**Country of origin**						
Native Portuguese	423 (93.0)	227 (95.8)	353 (95.7)	444 (94.9)	483 (94.3)	0.801
European non-Portuguese	7 (1.5)	1 (0.4)	3 (0.8)	6 (1.3)	6 (1.2)	
African	4 (0.9)	3 (1.3)	3 (0.8)	4 (0.9)	6 (1.2)	
South American	8 (1.8)	2 (1.8)	6 (1.6)	10 (2.1)	8 (1.6)	
Other	13 (2.9)	4 (1.7)	4 (1.1)	4 (0.9)	9 (1.8)	
**Parity and previous caesarean**						
Primiparous	275 (60.4)	142 (59.9)	243 (65.9)	316 (67.5)	329 (64.3)	0.142
Multiparous no previous caesarean	137 (30.1)	74 (31.2)	90 (24.4)	105 (22.4)	126 (24.6)	
Multiparous previous caesarean	43 (9.5)	21 (8.9)	36 (9.8)	47 (10.1)	57 (11.1)	
**Body mass index (Kg/m**^**2**^**)**						
<25.0	195 (42.9)	147 (62.0)	263 (71.3)	304 (65.0)	306 (59.8)	0.010
25.0 – 29.9	90 (19.8)	58 (24.5)	65 (17.6)	101 (21.6)	130 (25.4)	
> = 30	44 (9.7)	26 (11.0)	28 (7.6)	48 (10.3)	53 (10.4)	
no information (%)	27.7	2.5	3.5	3.2	4.5	
**Antenatal care**						
Only public services	328 (72.1)	146 (61.6)	184 (49.9)	209 (44.7)	321 (62.7)	<0.001
At least one visit at private services	121 (26.6)	74 (31.2)	183 (49.6)	255 (54.5)	137 (26.8)	
no information (%)	1.3	7.2	0.5	0.9	10.5	
Indications for induction*						
None	133 (29.2)	48 (20.3)	154 (41.7)	198 (42.3)	233 (45.5)	<0.001
One	199 (43.7)	124 (52.3)	161 (43.6)	188 (40.2)	185 (36.2)	
Two or more	120 (26.4)	59 (24.9)	49 (13.3)	74 (15.8)	83 (16.2)	
no information (%)	0.7	2.5	1.4	1.7	2.1	
**Foetal presentation**						
Cephalic	439 (96.5)	225 (94.9)	351 (95.1)	463 (98.9)	499 (97.5)	0.002
Non-cephalic	6 (1.3)	7 (3.0)	16 (4.3)	4 (0.9)	7 (1.4)	
no information (%)	2.2	2.1	0.5	0.2	1.2	
**Gestational age (weeks)**						<0.001
<37	9 (2.0)	9 (3.8)	26 (7.0)	30 (6.4)	13 (2.5)	
37	27 (5.9)	14 (5.9)	49 (13.3)	29 (6.2)	23 (4.5)	
38	70 (15.4)	36 (15.2)	109 (29.5)	84 (17.9)	46 (9.0)	
39	114 (25.1)	54 (22.8)	109 (29.5)	100 (21.4)	94 (18.4)	
40	140 (30.8)	48 (20.3)	68 (18.4)	116 (24.8)	300 (58.6)	
> = 41	95 (20.9)	76 (32.1)	8 (2.2)	109 (23.3)	36 (7.0)	
**Epidural**						
Yes	352 (77.4)	194 (81.9)	279 (75.6)	355 (75.9)	375 (73.2)	0.043
No	65 (14.3)	20 (8.4)	41 (11.1)	63 (13.5)	82 (16.0)	
no information (%)	8.4	9.7	13.3	10.7	10.7	
**Mode of delivery**						
Caesarean	161 (35.4)	77 (32.5)	150 (40.7)	214 (45.7)	248 (48.4)	<0.001
Vaginal	294 (64.6)	160 (67.5)	219 (59.3)	254 (54.3)	264 (51.6)	

* Conditions considered and counted were the usual major indications as explained in the Methods Section.

The main indications to proceed with surgical delivery were failed induction and/or poor progress of labour and foetal distress (87% of all caesarean deliveries after labour induction). Considering all women undergoing induced labour, the rates of failed induction and/or poor progress of labour were 21% and 27% for the women with no indications and those with at least one indication for induction, respectively. These proportions were significantly different across the hospitals and ranged between 13% and 28% (p = 0.007) in the former group of women and between 17% and 34% (p < 0.001) in the latter group. Rates of foetal distress demanding surgical delivery were 10% either among women undergoing induction without any indication or those with indicated induction. Such rate ranged across hospitals between 6% and 15% (p = 0.02) in the elective induction group and between 6% and 13% (p = 0.164) in the indicated induction group.

Table 3 shows the association between surgical delivery after induced labour and the hospital. There was a significant interaction between the hospital and the indication for induced labour (p < 0.01). We conducted a stratified analysis according to the presence of any indication for induced labour and controlled for parity and previous caesarean section and foetal presentation. Stronger differences between hospitals were observed among women who were induced without any indication. Compared to the hospital considered as reference, two hospitals presented significantly higher caesarean rates, the highest PR reaching 1.86 (95% CI, 1.33 – 2.62) among women without any indication for induced labour and 1.53 (95% CI, 1.12 – 2.10) among women undergoing indicated induction. The analyses restricted to primiparous, cephalic term pregnant group (n=1225) showed a similar pattern (Table 4). In this group none of the covariates modified the PRs of caesarean section after labour induction by hospital. The highest PR reached 2.06 (95% CI, 1.29 – 3.31) among women without any indication for induced labour and 1.61 (95% CI, 1.11– 2.34) among those undergoing indicated labour induction.

**Table 3:** Hospital differences in the risk of caesarean section after labour induction among all induced women (n = 2041)

	**With no indication for induction**		**With at least one indication for induction**	
	**% Caesarean**	PR* (95% CI)	**% Caesarean**	**PR‡ (95% CI)**
**Hospital**				
1	21.8	Reference	41.4	1.29 (0.94 – 1.76)
2	41.7	1.49 (0.83 – 2.70)	30.6	Reference
3	34.4	1.35 (0.86 – 2.13)	44.8	1.37 (0.99 – 1.91)
4	39.4	1.63 (1.06 – 2.49)	51.1	1.50 (1.10 – 2.05)
5	46.8	1.86 (1.23 – 2.82)	50.4	1.53 (1.12 – 2.10)
	p < 0.001	p = 0.035	p < 0.001	p = 0.065

* adjusted for foetal presentation and parity and previous caesarean.

‡ adjusted for parity and previous caesarean.

**Table 4:** Hospital differences in the risk of caesarean section after induction among primiparous cephalic term induced women (n=1225)

	**With no indication for induction**		**With at least one indication for induction**	
	**% Caesarean**	**PR (95% CI)**	**% Caesarean**	**PR (95% CI)**
**Hospital**				
1	26.8	reference	45.9	1.32 (0.90 – 1.94)
2	31.8	1.19 (0.51 – 2.78)	34.9	reference
3	38.8	1.45 (0.86 – 2.62)	44.9	1.29 (0.85– 1.95)
4	42.7	1.59 (0.97 – 2.62)	55.9	1.60 (1.10 – 2.34)
5	54.9	2.06 (1.29 – 3.31)	55.7	1.61 (1.11 – 2.34)
	p = 0.001	p = 0.022	p = 0.003	p = 0.077

## Discussion

The present study shows that public level III hospitals that have similar standards of care and provide free universal care to pregnant women presented different risk of caesarean delivery after induction. These differences were particularly evident in the absence of the foetal or maternal conditions that are usually considered indications for induced labour and remained after adjusting for multiple potential confounders.

Although a positive correlation between induction rates and caesarean rates at the hospital level has been reported [35], to the best of our knowledge no studies have addressed the differences between obstetric units for the risk of surgical delivery after induction, taking into account the potential differences in their case mixes.

It is well recognised that labour induction increases the risk of surgical delivery [10-19], but it is unclear whether such risk is avoidable. Major indications for labour induction, such as chronic or gestational hypertension [16,38] and diabetes [16,38-40] are themselves risk factors for caesarean section among women with spontaneous labour onset. These conditions also increase the likelihood of caesarean section when labour is induced, and the same is true for foetal growth restriction [41]. Furthermore, pregnancy duration beyond forty weeks increases the risk of longer labour, dystocia and foetal distress and, consequently, the risk of caesarean section as well [42]. Still, as maternal age [7] and BMI [43] increase, the likelihood of caesarean delivery also increases. This means that variation of case-mix across settings will lead to different caesarean rates. In our sample, there were differences across hospitals regarding conditions that are either indications for induction of labour or risk factors for caesarean section. Nonetheless, when we accounted for these factors, the risk of surgical delivery after induced labour remained different between the hospitals.

Our findings suggested disparities across hospitals at different levels of the management of induced labour, namely regarding the decision to proceed with surgical delivery. Failed induction (e.g., the inability to achieve the active phase of labour) is a reason pointed to perform a caesarean section [13,14], but there are no standardised criteria to diagnose it [44]. Instead, the definition of failed induction diverges across settings, regarding either the cervical status that marks the transition from the latent to the active phase of labour or the time-interval to consider that such transition failed, which variation is particularly evident, ranging between 8 and 48 hours [44,45]. The numbers of caesarean sections caused by failed labour will differ according to the definitions that are adopted in practice. Furthermore, induction has the potential to modify the normal progression of labour by increasing the duration among either primiparous [46,47] or multiparous women [15,46] who have an unfavourable cervical status at baseline. In such circumstances, it is difficult to define normal labour and to diagnose abnormally slow progression of labour that demands surgical delivery. This difficulty adds more variability to clinical judgement regarding the decision to proceed with surgical delivery after induction. In our sample, one fourth of the women with induced labour had a diagnosis of failed induction and/or poor progress in labour. Nonetheless, the proportion of such diagnosis was different across hospitals and was higher in the hospitals presenting also higher caesarean rates after induction.

Higher labour induction rates have been associated with increased caesarean section rates [35], most likely reflecting no appropriate selection criteria. This situation is particularly important in cases which there are no clear indication for prompt delivery. According to the obstetric guidelines, when no clear indication for induction is identified, the selection of women undergoing induction of labour should be based on favourability of cervix [20,22], and the use of cervical ripening agents should be considered when cervix is not favourable [22]. As a determinant of successful induction, the Bishop score has been commonly used to evaluate cervical status before induction, but there is a wide variation across settings regarding the cut-off point of this score to define a favourable cervix [45]. Different proportions of women undergoing induction with lower values for this score will determine the different caesarean rates. In the current study, there was not enough detailed information about cervical status at admission in the files to calculate a Bishop score. Although this issue prevented us from drawing conclusions concerning the appropriateness of practices, we observed striking differences across the five hospitals regarding the proportion of patients who underwent induction with any of the expected pregnancy or foetal characteristics that were considered indications. Indeed, this proportion was higher in the hospitals that had higher caesarean rates after induction. As the labour induction rates increased among the women with no clear indication for this procedure also the chances of selecting a woman with an unfavourable cervix who was at the greater risk of surgical delivery also increased [35]. Thus, our results suggested differences between hospitals regarding the selection criteria of women undergoing labour induction.

Our study suggested an institutional level risk of surgical delivery after induction, which emphasised the importance of local adherence to clinical protocols and policies regarding the selection of women and the management of induced labour. It has been previously reported that a physician effect exists in the risk of caesarean section after induction; this effect highlights the influence of individual clinical experience and practice in such risks [13]. Our findings could be the result of different hospitals having different number of physicians who are more prone to induce or deliver by caesarean. However, we are not dealing with private practices or an organisation based on individual doctors (our legal system demands the presence of at least two different doctors any time in the delivery room, and in large hospitals the number of obstetricians in charge often surpasses three); therefore such an explanation is implausible.

The main strength of this study is the large set of maternal, foetal and pregnancy characteristics evaluated that are both risk factors for surgical delivery and indications for labour induction. Thus, taking into account the variation in case-mix, we attempted to determine whether this variation was responsible for the differential caesarean rates that were observed between hospitals.

Our study was limited by the absence of information on the Bishop score at admission. Furthermore, the indications for induction were not always specified in the files. Although the proportion of such situations was similar in the five hospitals, we performed an exhaustive search of medical records for the presence or explicit absence of any foetal and pregnancy characteristics that were commonly considered indications for labour induction [20-22]. The final proportion of induced women with no indication for the procedure was calculated based on all available information. The guidelines used were provided by both ACOG and RCOG and covered the time period between 1999 and 2009.

Although epidural anaesthesia has been associated with an increased risk of caesarean section after induction [10,48], this factor cannot explain the differences that were observed across our hospitals. In spite of the hospital, the proportion of induced women receiving epidural anaesthesia was approximately 90% among those delivering vaginally and 85% if a caesarean section was performed. Additionally, the placement of an epidural catheter earlier in labour may increase the risk of surgical delivery [10]. In our sample there is no information about timing for epidural catheter placement, nor are any published data available describing the local practices. It is possible that differences could exist between hospitals regarding such obstetric practices but the final effect of these differences on the observed variability is most likely negligible.

## Conclusions

In conclusion, this study showed that the risk of caesarean section after induction varied significantly according to the hospital where the delivery occurred; these variations existed despite the differences in case mix, and the effect was particularly evident when there was no indication for induction. These findings suggest that the risk of surgical delivery after induction has an institutional level, emphasising the importance of local adherence to clinical protocols and policies to avoid unnecessary obstetric interventions.

## Competing interests

The authors declare no competing interests.

## Authors’ contributions

CT and HB conceived the study. CT and SC contributed to the data collection. HB was responsible for designing the cohort study. CT analysed the data and wrote the first draft of the manuscript. All of the authors contributed to the interpretation of results, commented on drafts and approved the final version of the manuscript.
